# An urban flood-risk assessment of South Jakarta, Indonesia: A methodological approach through frequency ratio, receiver operating curve and analytic hierarchy process

**DOI:** 10.4102/jamba.v18i1.1873

**Published:** 2026-01-26

**Authors:** Diana Puspitasari, Dyah R. Hizbaron, Estuning T.W. Mei

**Affiliations:** 1 Department of Disaster Management, Graduate School, Universitas Gadjah Mada, Yogyakarta, Indonesia; 2 Department of Environmental Geography, Faculty of Geography, Universitas Gadjah Mada, Yogyakarta, Indonesia; 3 Department of Development Geography, Faculty of Geography, Universitas Gadjah Mada, Yogyakarta, Indonesia

**Keywords:** urban, flood, susceptibility, vulnerability, risk, frequency-ratio, receiver operating characteristics, analytical hierarchical process

## Abstract

The main objective of this research is to conduct an urban flood-risk assessment approach for South Jakarta, Indonesia. Flood susceptibility was modelled using the frequency ratio (FR) method and validated with the receiver operating characteristic–area under the curve (ROC–AUC). Vulnerability was assessed using the analytic hierarchy process (AHP) across four domains: physical, economic, environmental and social. We used high-resolution spatial data (1:25 000) and a historical flood inventory to produce rapid, urban-scale risk information where conventional datasets are limited. The susceptibility map shows three classes: low, medium and high; the combined risk map indicates that most of the study area is at medium risk (108.64 km^2^, or 74.550%), low (37.08 km^2^ or 25.447%) and high (0.004 km^2^ or 0.003%). Vulnerability analysis identifies residential areas, critical infrastructure and key service zones as the most exposed elements. Susceptibility, vulnerability and risk are the three essential parameters incorporated into the spatial planning analysis to ensure comprehensive evaluation. The findings identify protection zones with particular risk levels, which require targeted mitigation strategies for any future development initiatives. Furthermore, this study highlights that the integration of FR, ROC–AUC and AHP provides reliable and operational flood-risk assessments that can be effectively incorporated into spatial planning and development policies in data-constrained urban settings.

**Contribution:**

This study presents an innovative and practical framework for urban flood-risk assessment, combining FR, ROC–AUC and AHP to evaluate flood susceptibility and vulnerability in South Jakarta, Indonesia. Leveraging high-resolution geospatial data at a detailed 1:25 000 scale, it addresses critical data gaps and equips policymakers with actionable tools to integrate risk sensitive strategies into urban spatial planning for further mitigation. The findings, revealing 74.55% of the area at medium flood risk, set a benchmark for advancing disaster resilience and sustainable urban development, offering valuable applications for other rapidly urbanising, data-constrained regions globally.

## Introduction

The main objective of this research is to evaluate the most suitable flood-risk analysis in South Jakarta, Indonesia. To do so, the research conducted a comprehensive review of methods, tools and techniques to pursue integrated urban flood-risk assessment. This research is grounded in the understanding that floods are the most frequent hazard affecting urban areas in South Jakarta, Indonesia. Urban flooding in South Jakarta is largely a consequence of rapid economic growth and intensive urban development, which has resulted in widespread impervious surfaces. The causes of recurrent annual flooding are complex and multifactorial. It has been further compounded by global drivers such as climate change and extreme weather events. Effective urban flood-risk management is therefore critical, both to adapt to climate change impacts and to achieve balanced land use and adequate drainage infrastructure (Kundzewicz et al. 2020). Urbanisation contributes to the rapid growth of urban populations, which puts pressure on the land and the environment and increases vulnerability and risk, resulting in immeasurable losses in the aftermath of disasters (Thoban & Hizbaron 2020). This implies that the impacts of flooding on vulnerable urban populations – such as low-income communities, informal settlements and residents in flood prone neighbourhoods – will continue to increase. Consequently, the overall flood-risk will rise if flood hazards are not effectively managed (Chimnonyerem, Ndukwe & Adannaya 2016).

Most recent research has predominantly focused on large-scale, riverine (fluvial) flooding, whereas urban pluvial flooding has been comparatively under-explored (Guo, Guan & Yu 2021; Kundzewicz & Pińskwar 2022).

Many urban areas, especially in developing countries, lack robust flood data, hindering effective flood-risk management, for example, South Jakarta, a city in the Special Province of Jakarta (or better known as Daerah Khusus Ibukota – DKI Jakarta). Capital of Indonesia is severely threatened by hydrometeorological disasters and climate change impacts such as extreme rainfall. Historical records indicate an increasing trend in flood frequency in the study area, including the devastating flood with an extensive impact in January 2019 (Drestalita & Saputra 2019). As a designated national strategic development area, South Jakarta has been designed to accommodate high carrying capacity, advanced infrastructure and services and intensive vertical and horizontal development. However, this rapid expansion has also heightened the city’s flood risk.

Government Regulation No. 26 of 2008 on the free-runoff policy requires that any construction activities in intensely developed areas must adhere to restrictions to limit resulting water runoff. The regulation mandates that for each construction site, drainage channels or infiltration zones should be designed with the dimension or capacity that accommodates runoff discharge to prevent inundation and flooding (Deliana 2016; Indratmoko 2010). Using such an approach in infrastructure planning highlights the need for more detailed flood-risk information to support both public and private sector decision-making in South Jakarta.

To apply the free-runoff principle, South Jakarta requires an integrated flood-risk analysis in its detailed spatial plan and also its infrastructure planning. The provincial government has introduced measures to reduce flood risk, increase preparedness and implement emergency responses using several regulation documents, such as spatial planning, infrastructure planning and contingency planning. However, incorporating flood-risk analysis based on the urban and/or city level remains a great task to complete because of lack of available data and methodological hurdles (Hizbaron et al. 2012). The incorporation of flood-risk specific components into urban or spatial planning is continually evolving. Recent research emphasises the need for risk-sensitive land-use planning, demonstrating how spatial design can reduce urban flooding susceptibility (Liu et al. 2020). The literature on the integration of flood-risk modelling into spatial planning is expanding, using spatial-statistics modelling, participatory analysis or policy-based observations.

Nonetheless, problems remain in tailoring these models to specific local contexts and ensuring that policy frameworks incorporate them effectively.

Susceptibility and vulnerability have been examined as separate components, and risk analysis has largely focused on calculating a composite flood-risk index rather than producing integrated spatial information (Sutanta, Rajabifard & Bishop 2010) as shown from the Indonesian case. The Indonesia Disaster Risk Index (IRBI) of the National Disaster Management Agency comprises three components of risk: hazards, vulnerability and regional capacity (BNPB Head Regulation Number 2 of 2012 on General Guidelines for Disaster Risk Assessment, Indonesian Government Regulation Number 26 of 2008 on Spatial Plans). The IRBI was calculated from hazard and vulnerability analysis dated in 2013 at national level (1:250 000), and only regional capacity details are updated annually (Badan Nasional Penanggulangan Bencana 2020), with no adequate guideline on flood-risk analysis at urban and/or city level. The integration of components of urban flood-risk analysis into the spatial plan is normally taken from the large scale as presented by the national-level risk, then downscaled into the urban/city level. The accuracy of the spatial-statistics modelling applied in the Indonesian case is limited by the lack of updated data, scale differences and inflexible temporal consistency of the spatial planning and risk information generated (Kim & Newman 2020; Youssef, Pradhan & Sefry 2016).

Currently, remote sensing and geographic information systems (GIS) are widely used methods to overcome shortages in data availability and processing, especially to generate updated spatial-statistical flood-risk model.

Acknowledging their significance has led to a new discourse in flood studies. Flood hazard, vulnerability and risk mapping processes now involve geospatial data, often with multi-temporal resolution (Youssef et al. 2016), and allow the detailed urban and/or city level to get free-access data through the Google Earth Engine (GEE) for example. Geographic information system is a tool to bridge the processing, management, presentation and integration of different spatial databases, such as historical data, causal factors and flood-risk elements. Using spatial data from the GEE, combined with GIS-based flood susceptibility, vulnerability and risk modelling, it can generate the expected outcome at urban and/or city level (Hizbaron et al. 2012).

Flood risk is typically assessed by combining susceptibility and vulnerability maps with GIS-based models widely used to perform this integration. For example, data on flood susceptibility components can be entered into applications such as HAZUS-MH, a standardised, GIS-based hazard-loss estimation tool developed by the Federal Emergency Management Agency (FEMA), to model the potential extent and spatial distribution of flooding. However, this method suffers from inaccuracies (high error values) for areas with complex topography. To reduce inaccuracy, 2D models like rainfall-runoff inundation (RRI) and Hydrologic Engineering Centre-River Analysis System (HEC-RAS) are employed, but these methods entail comprehensive and detailed information, such as river cross sections, soil infiltration rates and meteorological records, which creates problems in meeting data requirements for the modelling (Cea & Costabile 2022; Costabile & Macchione 2015; Dewan 2013). Popular statistical models with straightforward processes and analysis for flood susceptibility mapping include the logistic ratio (LR), weight of evidence (WoE) and frequency ratio (FR) (Rahmati, Pourghasemi & Zeinivand 2016; Tehrany, Pradhan & Jebur 2014). Frequency ratio is a bivariate statistical method used to determine comprehensive flood-conditioning factors in an area and shows good accuracy in predicting flood susceptibility (Samanta et al. 2018; Tehrany & Kumar 2017). In addition, FR models non-linear relationships between flood occurrences and their causing factors (Siahkamari et al. 2018). Several complex factors contribute to flooding in urban regions in South Jakarta, which, with good historical data, can be quickly modelled using FR to obtain the distribution of flood-prone areas. Now, the remaining question is how to incorporate such models into the spatial planning for sustainable urban management.

Flood vulnerability components are often modelled using GIS and multidimensional approaches to identify at-risk elements (Meyer, Haase & Scheuer 2009; Nasiri & Kalalagh 2013). In recent years, studies have used multiple-criteria analysis (MCA) for this modelling to determine vulnerability factors more comprehensively and reliably (Tay & Sreevalsan-Nair 2023), including breaking down and then incorporating the complexity of at-risk elements (Mahmoud & Gan 2018). The analytic hierarchy process (AHP) is an example of a multi-criteria approach that can be used to model vulnerability, particularly in the case of urban flooding, because it works with limited data availability and is able to incorporate spatial planning elements into the modelling (Ouma & Tateishi 2014; Wang et al. 2011). Flood vulnerability and spatial planning intersect through their shared concern with elements at risk, including land use, population and infrastructure. Because these components are inherently exposed to flooding, vulnerability analysis should be treated as a critical layer that can be incorporated into spatial plans once flood risk has been evaluated.

In this research, urban flood risk is defined as the combined outcome of susceptibility and vulnerability assessments. Susceptibility was analysed using the FR model, while vulnerability was evaluated using the AHP. Integrating these two approaches enables assessment of the severity of urban flood risk in a form that is compatible with the detailed spatial plan of Jakarta. The central aim of this study is to develop a practical modelling framework for urban flood-risk assessment that provides detailed information for spatial planning. The completed research is expected to generate a 1:25.000-scale risk map aligned with planning requirements. Once completed, this map can be directly incorporated into spatial plans to support development policy, disaster mitigation strategies and the protection of communities and assets.

## Research methods and design

### Study area

South Jakarta is an economic hub of Indonesia’s capital, characterised by advanced infrastructure, extensive services and intensive horizontal and vertical development. Morphologically, it occupies alluvial plains composed mainly of tuff and clay. With clay occurring widely in the city, water rarely infiltrates (vertical) but instead flows on the ground (horizontal). The city has varying elevations from 5 m to 50 m above mean sea level (masl). However, it is mostly below 12.5 masl (covering 87.13% of the total area) or low-lying land with a slope gradient of 0% – 8% (flat to sloping terrain). These geographical and morphological profiles show the area’s high proneness to floods because runoff collects and accumulates quickly and prevents prompt infiltration into the soil.

The main drainage network of South Jakarta consists of the Ciliwung, Krukut, Pesanggrahan, Kali Grogol and Kali Baru Barat Rivers. On average, the city receives 2001 mm – 2500 mm of rainfall per year. Based on the pattern from 2013 to 2021, floods occur from November until March, peaking in January and February. A previous study revealed that the monthly average rainfall from December until March is always above 200 mm (Ginting 2015). The city lies in the upstream portion of Jakarta and is traversed by several rivers, which makes it highly prone to inundation during periods of extreme rainfall.

### Data availability

The primary and secondary data used in the research are summarised in Table 1. The primary data were collected through surveys and field observation. The secondary data were derived from satellite images, geospatial databases, statistical information from databases of responsible agencies, previous studies and regulations (Sugiyono 2015). The primary data are used to describe actual conditions in the field and validate current land-use types, and the secondary data were publicly accessible spatial data used as the basis for building the integration of flood-risk and land-use plans. All parameters were processed at a scale of 1:25 000 to match the spatial plan.

**TABLE 1:** Sources and specifications of the research data.

No	Data	Data sources	Type, scale, or resolution	Extracted data
1	Indonesia Digital Elevation Model (DEM Nasional)	Geospatial Information Agency (BIG)	8.3 m	Elevation Slope gradient Slope aspect Curvature Topographic wetness index (TWI)
2	Rainfall	Weather stations of the Meteorology, Climatology, and Geophysics Agency (BMKG) and CHIRPS data	Rainfall data from 2012 to 2021	Rainfall distribution
3	Drainage network	Indonesia Topographic Maps (RBI) by Geospatial Information Agency (BIG)	1:25 000	Distance from river
4	Flood occurrences	Regional Disaster Management Agency (BPBD) for DKI Jakarta	Flood area by district from 2013 to August 2021	Flood distribution (map)
5	Satellite image	SPOT 6/7	6 m	Land-use zoning based on the detailed spatial plan document
6	Land-use	Jakarta Satu (Online Data Centre run by The Regional Government of DKI Jakarta)	1:25 000	
7	Actual land-use condition (for validation)	Field survey with GPS devices and cameras to create a photo library	1:25 000	Points of validation to check the actual land-use condition
8	Demography	BPS-Statistics Indonesia and Village Potential Statistics (PODES) of South Jakarta City in 2021	Sub-district level	Population density distribution Sex ratio (%) Age-vulnerable population (%) Persons with disabilities (%) Economically disadvantaged population (%)
9	Number of houses	Jakarta Satu and PODES 2021 from Central Bureau of Statistics (BPS)	Sub-district level	Number of houses
10	Road network	Jakarta Satu	1:25 000	Percentage of road length based on road type
11	Documents and regulations on disaster and spatial planning	Laws, national and regional regulations, and technical documents	National, regional, provincial, and city levels	GIS integration aspects

GPS, global positioning system; GIS, geographic information system.

### Modelling steps

**FIGURE 1:**
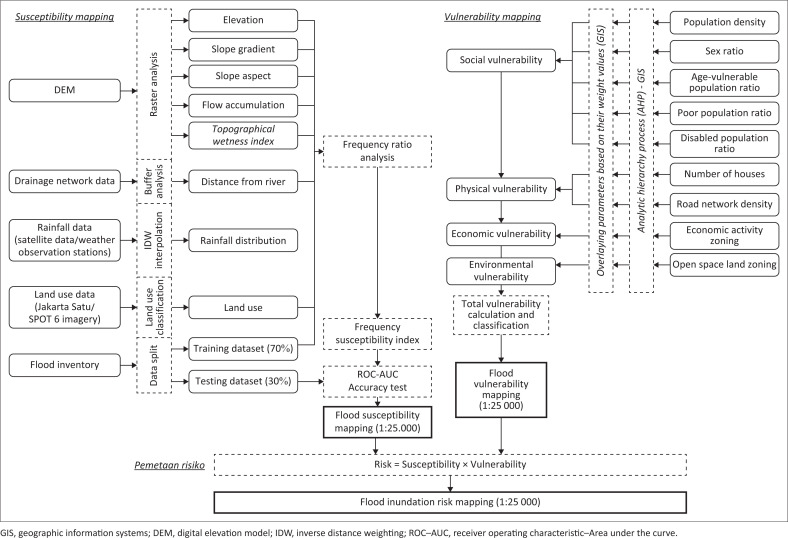
Flood-risk modelling flowchart.

In this study, the GIS was used to compile, visualise and analyse numerous layers of geospatial data, including topography, land use, rainfall patterns, soil type, drainage network, elevation, etc. The GIS provides a platform for combining different forms of spatial data and performing MCA. In this context, the GIS was used to handle the spatial data inputs required by the FR and AHP models, as well as to generate risk of flood maps based on the models’ weighted outputs (Figure 1).

### Susceptibility mapping with frequency ratio model

The FR model is a bivariate statistical analysis that can model the spatial distribution of flood events based on multiple conditioning factors. Frequency ratio model is a statistical method commonly used in flood-risk assessment to identify the link between previous flood occurrences and relevant elements (e.g. elevation, slope, rainfall). The FR model assumes that future floods will follow patterns similar to previous flood disasters (Rahmati et al. 2016; Ramesh & Iqbal 2022; Samanta et al. 2018; Tehrany et al. 2014). The FR model is useful for flood susceptibility modelling as it is simple and can handle spatial data. It assesses the possibility of flooding by comparing the presence of specific causal variables in areas with known flooding events to areas without flooding, resulting in a clear probability ratio.

**FIGURE 2:**
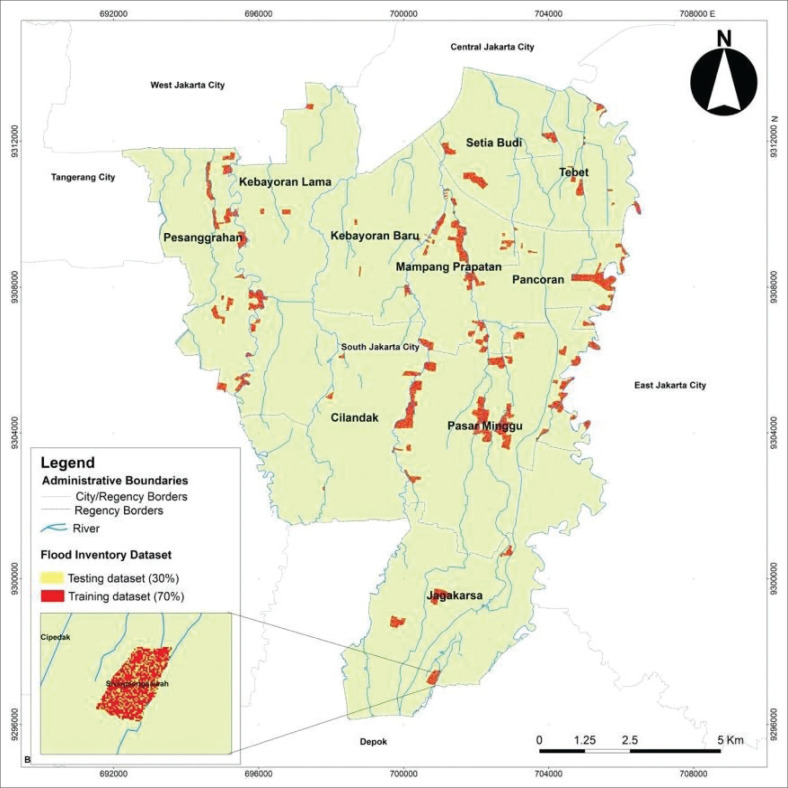
Sample distribution of historical data on flood events.

For each factor, the historical flood locations were mapped, and the ratio between flood-prone and non-flood-prone areas for each factor category was calculated. A correlation analysis was performed to determine their relationship with floods occurrence. A greater bivariate probability value (more than 1) indicates a strong correlation between flood occurrences and their conditioning factors, whereas a low value (less than 1) suggests a weak correlation (Youssef et al. 2016). Based on historical data, the spatial distribution of flood events in South Jakarta is shown in Figure 2.

According to the flood inventory dataset, 30% (22 304 flood pixels) of the data was treated as the testing dataset, while the remaining 70% (74 347 flood pixels) of the dataset is treated as the training dataset. The spatial distribution of flood events was derived from the existing dataset, which records the number of neighbourhood units exposed to flooding. The dataset was randomly divided into training and testing subsets.

The flood inventory data were then evaluated against seven conditioning factors. Seven conditioning factors representing topography, hydrology and human activities were used for the FR-based flood susceptibility mapping: topography (elevation, slope gradient, slope aspect and TWI), hydrology (rainfall and distance from the river) and human activities (land-use type and zoning based on spatial plan documents), as depicted in Figure 1 (Dottori et al. 2015; Nasiri et al. 2019; Nkwunonwo et al. 2016; Papaioannou et al. 2016). The first step was to calculate the FR value for each factor on the training dataset using the *tabulate area* and *raster calculator* tools in ArcGIS. Equation 1 was used in the calculation (Rahmati et al. 2016; Siahkamari et al. 2018):


$$FR=\frac{Number\;of\;flood\;pixels\;in\;each\;factor/total\;flood\;pixels}{Total\;pixels\;in\;each\;factor/total\;pixels\;of\;the\;entire\;area}$$ [Eqn 1]

Then, FR was mathematically normalised into relative frequency (RF) with a value in the range of 0 to 1 using Equation 2 (Youssef et al. 2016). Relative frequency was used to determine the correlation between each class of a conditioning factor and flood using a predictor rate (PR). Predictor rate acted as the weight value of each factor obtained with Equation 3. Both RF and PR were calculated in Microsoft Excel:


$$RF=\frac{FR\;of\;each\;class\;in\;each\;factor}{Total\;FR\;of\;all\;classes}$$ [Eqn 2]

$$PR=(RF_{max}-RF_{min})/{\left(RF_{max}-RF_{min}\right)}_{min}$$ [Eqn 3]

Flood Susceptibility Index (FSI) was assessed by summing a series of multiplication results of the weight (PRi) and the RF value of each factor (RFi) for the total number of the considered factors (*n* = 7), as formulated in Equation 4. Afterward, FSI values were categorized into three classes of susceptibility: low, medium, and high, and then mapped in ArcGIS 10.3:


$$FSI=\sum_{i=1}^{n}PR_{i}\times RF_{i}$$ [Eqn 4]

### Model validation

The ROC–AUC method is an effective tool for assessing the effectiveness of predictive models, especially in flood susceptibility assessments (Lin, Wu & Liang 2019). It is a frequently accepted technique for evaluating the validity of binary classification models, such as flood-risk predictions, by comparing predicted flood-prone areas to actual flood occurrences (Fawcett 2006). In this work, the ROC–AUC method was used to test the accuracy of flood susceptibility models created using the FR methodologies. The ROC–AUC approach was chosen because it provides a convenient and quantitative way to assess model performance, providing that both models are evaluated not only for technical execution but also for practical applicability in flood susceptibility mapping.

The modelling results were validated by compiling a no-flooding dataset equal in size to the testing dataset.

No-flooding data were placed randomly within non-flooded areas using Create Random Points tool in ArcGIS.

Two datasets were created using the flood history data: 70% (training data, 74 347 flood pixels) were used to build the susceptibility mapping model, and 30% (testing data, 22 304 flood pixels) were used to assess the model’s prediction accuracy. Afterward, the no-flooding and testing data were combined and classified into ten classes to facilitate the mathematical operation of the validation. The values of the classified dataset at the randomly created points were extracted using the ArcGIS tool *extract values to points* and then calculated in Microsoft Excel to process the model validation.

The flood susceptibility map was evaluated using the ROC–AUC method, comprising the receiver operating characteristic (ROC) curve and the area under the ROC curve. The area under the curve was used to assess the prediction accuracy and model development quality (Hong et al. 2017), and the ROC curve determined the model’s level of success in predicting flood-prone areas based on previous flood events (Siahkamari et al. 2018). The ROC curve was generated by plotting *true positive rates* (TPR/*sensitivity*), that is, location points of flood events, against *false positive rates* (FPR/1-*specificity*), that is, location points of predicted floods (Ramesh & Iqbal 2022). The qualitative relationship between AUC and prediction accuracy can be classified into five categories: poor or unsatisfactory (0.5–0.6), fair or satisfactory (0.6–0.7), good (0.7–0.8), very good (0.8–0.9) and excellent (0.9–1) (Rahmati et al. 2016).

### Vulnerability modelling with analytic hierarchy process

Urban flood vulnerability encompasses physical, social, economic and environmental vulnerabilities. The four parameters explain elements at risk of damage, loss and destruction in the event of urban flooding. The parameters and variables used in the assessment are summarised in Table 2. The spatial distribution of social vulnerability was investigated with the district (administrative boundaries) as the unit of analysis. The AHP approach in the vulnerability research module was performed in the ArcGIS environment in three stages. The first stage was to classify variables for every vulnerability parameter and rank their classes. Secondly, the weight value of each variable was assessed with the AHP approach and calculation. Thirdly, the four variables were calculated as total vulnerability using the overlay analysis in ArcGIS and then classified into low, medium or high.

**TABLE 2:** Frequency ratios of flood-conditioning factors in South Jakarta.

Conditioning factor	Class	Class pixel	Class pixel (%)	Flood pixel	Flood pixel (%)	FR
Slope gradient (^o^)	0–2	288 774	13.772	57 974	13.491	0.980
	2–4	599 305	28.581	115 932	26.979	0.944
	4–8	846 591	40.374	176 779	41.138	1.019
	8–16	318 309	15.180	71 223	16.574	1.092
	16–35	43 609	2.080	7811	1.818	0.874
	35–55	260	0.012	0	0.000	0.000
Elevation (masl)	0–10	52 234	2.475	15 538.66	3.616	1.461
	10–20	519 017	24.588	15 3612.9	35.747	1.454
	20–30	638 371	30.243	15 3962.6	35.829	1.185
	30–40	390 494	18.500	71 847.57	16.720	0.904
	40–50	248 001	11.749	6228.788	1.450	0.123
	50–60	158 188	7.494	18 070.15	4.205	0.561
	> 60	104 507	4.951	10 459.03	2.434	0.492
Slope aspect	North	598 498	28.543	119 437.8	27.79436	0.974
	East	507 520	24.204	115 690.6	26.92233	1.112
	South	517 886	24.698	102 083.8	23.75591	0.962
	West	472 944	22.555	92 507.49	21.5274	0.954
TWI	0–6.0	1 170 052	55.815	229 582	53.426	0.957
	6.0–6.5	166 742	7.954	33 192	7.724	0.971
	6.5–7.0	129 685	6.186	25 207	5.866	0.948
	7.0–7.5	97 027	4.628	19 411	4.517	0.976
	7.5–8.0	67 039	3.198	13 923	3.240	1.013
	8.0–8.5	52 273	2.494	10 967	2.552	1.023
	> 8.5	413 488	19.725	97 437	22.675	1.150
Distance from river (m)	0–10	30 349	1.447	18 761.31	4.364	3.016
	10–30	60 646	2.892	37 988.94	8.837	3.056
	30–50	59 365	2.831	34 308.3	7.981	2.819
	50–100	139 959	6.674	69 524.26	16.172	2.423
	> 100	1 806 710	86.156	269 311.8	62.646	0.727
Rainfall (mm/year)	1900–2000	1955	0.093	0	0.000	0.000
	2000–2100	365 888	17.448	53 510.95	12.447	0.713
	2100–2200	353 787	16.871	79 833.41	18.570	1.101
	2200–2300	284 815	13.582	80 366.35	18.694	1.376
	2300–2400	392 334	18.709	129 480.5	30.119	1.610
	2400–2500	196 056	9.349	51 387.5	11.954	1.279
	2500–2600	172 722	8.237	5096.281	1.185	0.144
	2600–2700	246 165	11.739	22 425.3	5.216	0.444
	2700–2800	83 307	3.973	7794.312	1.813	0.456
Land-use zoning (Zones allocated for single/ multiple purposes)	Multipurpose zones	11 084	0.590	1432	0.370	0.626
	Arable green zones	1170	0.062	0	0.000	0.000
	Urban forests	11 821	0.630	0	0.000	0.000
	Industrial and trade zones	12 926	0.688	2615	0.675	0.980
	Green belts	123 837	6.595	33 709	8.698	1.319
	Special purpose zones	22 264	1.186	2315	0.597	0.504
	Public and social services	106 818	5.689	17 904	4.620	0.812
	Cemeteries and burial grounds	25 658	1.367	1907	0.492	0.360
	Government offices	34 290	1.826	15 139	3.906	2.139
	Others	113 789	6.060	20 610	5.318	0.878
	Aquaculture	7296	0.389	4438	1.145	2.947
	Offices, trade and services	245 346	13.067	39 679	10.239	0.784
	Residential zones	1 152 736	61.394	247 403	63.840	1.040
	Blue spaces	8575	0.457	383	0.099	0.216

TWI, topographic wetness index.

### Risk mapping

This integrated approach combines historical data (through the FR method) and expert judgement (via the AHP), enabling a more comprehensive analysis of urban flood risk. It is particularly suitable in contexts with limited data availability or complex planning requirements. The urban flood-risk levels were determined and mapped by combining and integrating susceptibility and vulnerability assessments (Dandapat & Panda 2017), as presented in Equation 5 (Apel et al. 2009).


Risk (R)=Susceptibility (H) x Total Vulnerability (V) [Eqn 5]

The susceptibility and vulnerability maps transformed into raster data were spatially overlaid and used as the input for risk index calculation with the *raster calculator* tool (Abdrabo et al. 2020). Consequently, the risk assessment results contain maps of urban flood susceptibility factors and physical, social, economic and environmental vulnerability factors. Risks were classified into three levels: low, medium and high. Fuzzy membership was used to normalise the spatial data in flood susceptibility, vulnerability and risk assessments, resulting in a homogenous index range of 0–1.

### Ethical considerations

This article followed all ethical standards for research without direct contact with human or animal subjects.

## Results

### Quality and resolution of the spatial data

The quality and resolution of spatial data are vital factors in flood-risk assessment, especially when using GIS-based models such as the FR and AHP. Those variables have a substantial impact on the accuracy, reliability and application of the results (Chen et al. 2005). The quality of spatial data is determined by its accuracy, consistency and relevance to the studied area. High-quality spatial data should accurately reflect real-world conditions, with minimal errors, as flaws may influence flood hazard predictions and mislead decision-makers (Bilskie et al. 2014; Sanyal & Lu 2005). This research uses the type, resolution and data source of each variable in Table 1 to produce a modelling scale used in regional planning at a scale of 1:25 000. This scale was chosen because it is a mapping need according to the applicable regulations in Indonesia.

### Flood susceptibility mapping with frequency ratio

Frequency ratio was calculated for each class of each conditioning factor (Table 2), in which FR > 1 indicates a strong correlation between flood and a particular class, and PRs were calculated to assign weight values to these factors, as depicted in Figure. The slope gradient of 8–16° was found to have the highest RF of 1.092, indicating a higher flood frequency at the foot of slopes compared to other terrains (Tehrany & Kumar 2017). Similarly, the correlation between elevation and flood frequency showed FR > 1, indicating a higher probability of occurrence in low-lying areas (Khosravi et al. 2016).

The topographic parameters or terrain attributes are strongly related to flood events, as evident from their PRs. However, the correlation between slope aspect and floods did not significantly differ across the classes. The slightly varied FRs resulted from a homogenous slope aspect that is distributed relatively homogenously with similar area percentages throughout South Jakarta (see Table 2 and Figure 3). The slope aspect also had a low PR compared to other conditioning factors; hence, it can be concluded that it is a terrain attribute with less significant control over flood occurrences in the study area (Figure 4). The same case applies to the secondary topographic parameter, topographic wetness index (TWI). Topographic wetness index showed no substantial difference between the FR values of its classes and had the lowest PR of 1.186 among the conditioning factors because South Jakarta has a homogenous relief (Figure 4). These results indicated a direct correlation between soil moisture and flooding (Samanta et al. 2018).

**FIGURE 3a:**
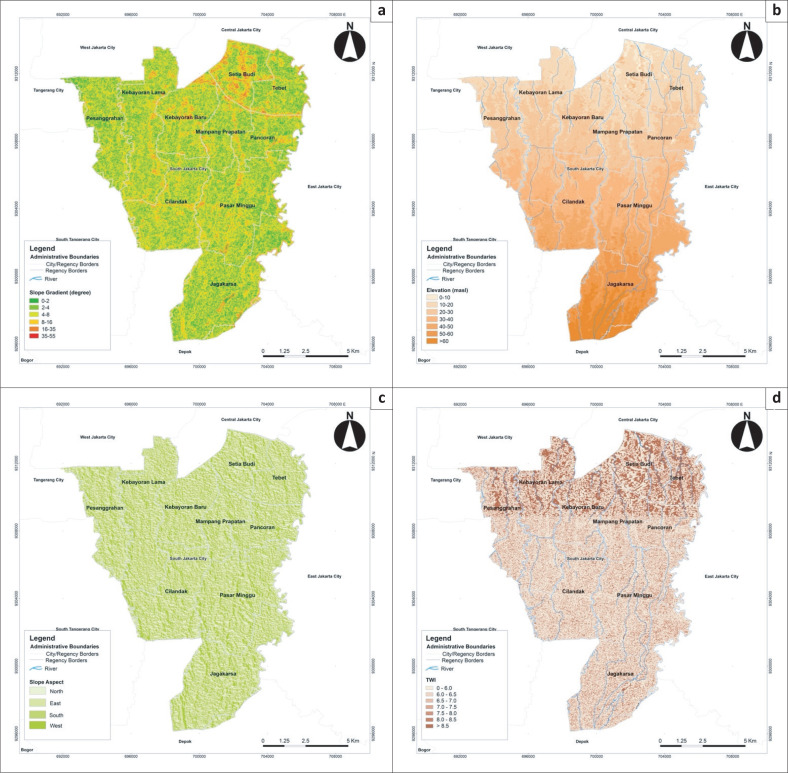
Flood susceptibility parameters: (a) slope gradient, (b) elevation, (c) aspect, (d) topographic wetness index.

**FIGURE 3b:**
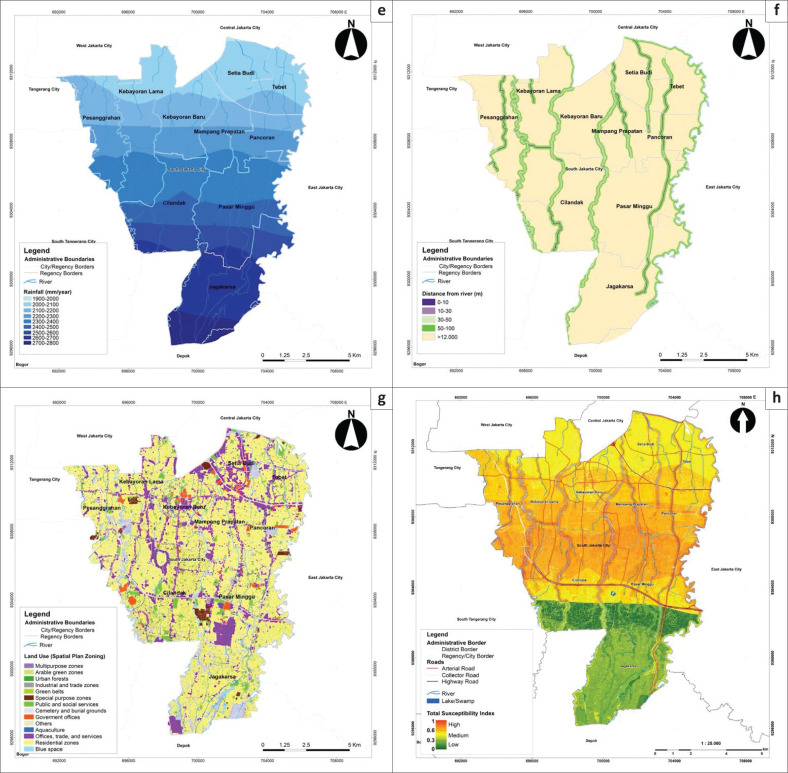
Flood susceptibility parameters: (e) rainfall, (f) distance from the river, (g) land-use and (h) Flood susceptibility map of South Jakarta.

**FIGURE 4:**
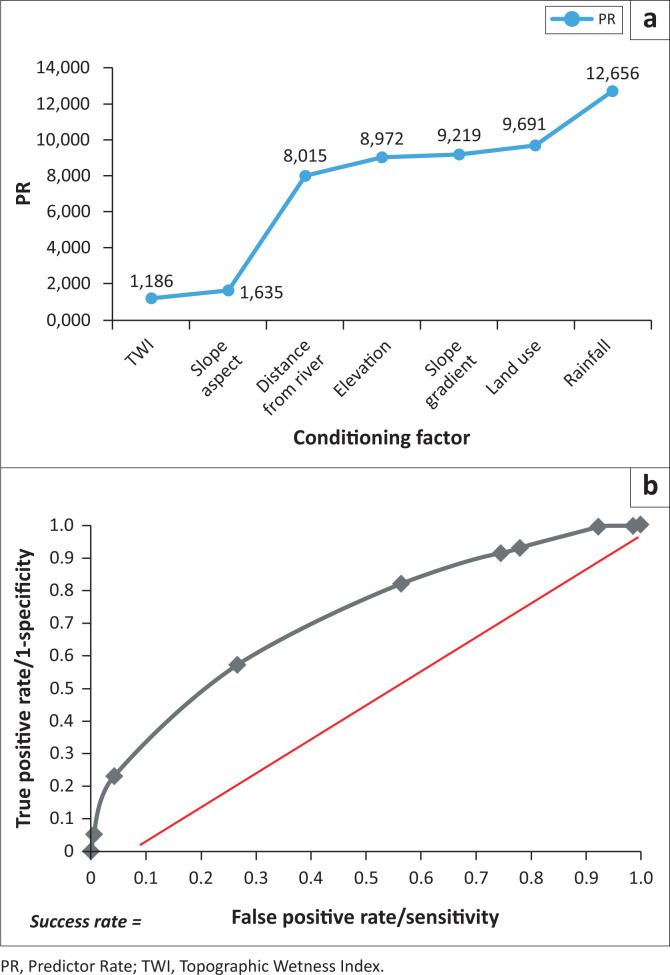
Predictor rates of each conditioning of flood susceptibility factors and receiver operating characteristic curve showing the success rate of the frequency ratio model.

The amount of rainfall (FR > 1) is shown as a major determinant of flood formation (Sarkar & Mondal 2020).

Rainfall had a PR of 12.566, making it the most significant conditioning factor of flooding (Figure 4). Therefore, extreme rainfall in Ciliwung, Pesanggrahan and Krukut watershed systems is always an indication of flooding in the city. Similarly, distance from river networks had a PR higher than 1 for all its classes, except the 100 m distance. Areas located closer to the river had a higher FR. The probability of flooding is higher with a shorter distance to the river (Kabenge et al. 2017). In addition, this factor had a high PR, or weight value, indicating a strong determinant.

Land use plays an essential role in identifying areas prone to flooding (Samanta et al. 2018). Land use identification helps assess the extent of impervious surfaces, which contribute to increased surface runoff in urban areas (Al-Zahrani, Al-Areeq & Sharif 2017). An extensive impenetrable surface accelerates runoff velocity and thus has the highest susceptibility to flooding (Ullah & Zhang 2020). Land use had the highest PR in areas associated with bodies of water, that is, aquaculture, and green belts on river banks (Figure 4). Moreover, built-up lands had high FRs, especially government offices and residential zones (FR > 1). In terms of area, residential zones are the type of land use most widely associated with floods because up to 63.84% of the total housing area had a high probability of flooding. Land use, as a conditioning factor of susceptibility, showed the second-highest PR, indicating a strong relationship between urban flooding and land use patterns (Figure 4). Built-up areas near river networks tend to be the most susceptible to flooding, mainly because they generally have a large population size and are centres of economy, services and infrastructure (Khosravi et al. 2016; Sarkar & Mondal 2020).

Prior to mapping, the FSI was normalised using an ArcGIS geoprocessing tool called *fuzzy overlay* to create a scale of 0–1 to match the risk and vulnerability index range. Therefore, the derived index values were transformed from 193.399–903.571 to 0.0534–0.8085. The FSI was later classified into low, medium and high susceptibility using the equal method, according to Head of National Disaster Management Agency Regulation No. 2 of 2012. Figure 3(h) indicates that South Jakarta is exposed to three classes of susceptibility: low (22.451%, 28.845 km^2^), medium (73.171%, 94.101 km^2^) and high (54.377%, 5.624 km^2^). Areas highly susceptible to flooding were characterised by high rainfall, settlements or other types of buildings close to rivers and flat to gently sloping terrain. The map also shows that these areas were allocated for residential purposes, government offices and economic facilities and services and situated along the river channels (see Figure 3). Meanwhile, low susceptibility was primarily attributed to elevation, that is, more than 40 m.

The developed FR model was tested for its quality and accuracy of predictions using the ROC curve (Samanta et al. 2018; Sarkar & Mondal 2020; Ullah & Zhang 2020). The ROC curve plots (Figure 4) *false positive rates* on the x-axis against *true positive rates* on the y-axis according to the FR analysis. The prediction’s accuracy was assessed using the area under the ROC curve (AUC). The model cannot be used to predict and map flood susceptibility if the AUC is less than or equal to 0.5, but the predictions are accepted if the AUC is higher than 0.75 (Saha et al. 2021; Sarkar & Mondal 2020), as shown in Figure 4. The flood inventory (historical data) was divided into: 70% (training data, 74 347 flood pixels) to build the model for susceptibility mapping and 30% (testing data, 22 304 flood pixels) to test for the accuracy of the model’s prediction. The accuracy test produced an ROC curve with a success rate of 0.798 (Figure 4), indicating that the FR-based flood susceptibility map for South Jakarta has good predictive performance and can be applied to estimate flood trends as well as to support further analyses such as flood-risk assessment.

### Vulnerability mapping with analytic hierarchy process

The total vulnerability index was calculated from four weighted parameters: social, physical, economic and environmental vulnerability. Each parameter consisted of variables that were ranked based on expert-defined criteria and analytical considerations. Social and environmental vulnerability variables were ranked according to the Head of National Disaster Management Agency Regulation No. 2 of 2012, which explains the social conditions of people exposed to disaster impacts and the environmental characteristics of an area prone to being severely affected. For the physical vulnerability, the variables were ranked by considering the type and level of physical infrastructure in urban areas that would suffer losses in the event of a flood. As for the variables of economic vulnerability, the ranks were determined by assessing financial losses indirectly from potentially affected urban economic activities (i.e. land-use type) based on gross domestic product (GDP) in current prices in South Jakarta (Table 3).

**TABLE 3:** Ranks and weights assigned to each class of each parameter in total flood vulnerability.

Parameter	Variable	Class	Rank	Weight	CR
Social vulnerability	Population density (people/ha)	< 5	1	0.332	1.32%
		5–10	2	-	-
		> 10	3	-	-
	Sex ratio (%)	> 40	1	0.110	-
		20–40	2	-	-
		< 20	3	-	-
	Age-vulnerable population ratio (%)	< 20	1	0.186	-
		20–40	2	-	-
		> 40	3	-	-
	Disabled population ratio (%)	< 20	1	0.186	-
		20–40	2	-	-
		> 40	3	-	-
	Poor population ratio (%)	< 20	1	0.186	-
		20–40	2	-	-
		> 40	3	-	-
Physical vulnerability	Number of houses (per district)	638–5506	1	0.75	0.01%
		5507–10 375	2	-	-
		10 376–15 244	3	-	-
	Road network density (km/km^2^)	0.157–12.699	1	0.25	-
		12.700–25.242	2	-	-
		25.243–37.784	3	-	-
Economic vulnerability	Aquaculture	-	1	0.016	7.88%
	Arable green zones	-	2	0.016	-
	Industrial and trade zones	-	3	0.060	-
	Government offices	-	4	0.079	-
	Special purpose zones	-	5	0.093	-
	Public and social services	-	6	0.157	-
	Offices, trade and services	-	7	0.266	-
	Multipurpose zones	-	8	0.313	-
Environmental vulnerability	Blue spaces	-	1	0.039	5.58%
	Others (shrubs/empty lands)	-	2	0.109	-
	Green belts	-	3	0.160	-
	Cemeteries and burial grounds	-	4	0.244	-
	Urban forests	-	6	0.448	-

CR, Consistency Ratio.

Analytic hierarchy process assigned a relative weight value to each parameter and variable. The social vulnerability was given the largest weight, 40%, because it represents the most variable exposed to flooding, population characteristics (Bigi et al. 2021). Meanwhile, the physical and economic vulnerability was 25% each, and the environmental vulnerability was 10%. Land-use zoning was used to capture the significance of urban economic activities and measure potential financial losses.

The variable overlay analysis with fuzzy membership resulted in a total vulnerability index ranging from 0.182 to 0.670. The distribution of total vulnerability levels is mapped in Figure 4. Overall, most of the South Jakarta area had medium (47% of the study area) and low vulnerability (53%). The total vulnerability showed a similar spatial pattern to the physical and social vulnerability. The medium vulnerability was observed in areas where the distributions of the number of houses and population densities overlapped, particularly in the districts of Pesanggrahan, Kebayoran Lama, Cilandak, Pasar Minggu and Jagakarsa. These results correspond to existing land-use conditions in South Jakarta, where the majority (61%) of the area is used for residential zones.

The AHP is a decision-making process that prioritises factors based on expert assessment. In flood-risk assessment, AHP assists in determining the relative relevance of various flood-related factors. Analytic hierarchy process helps the methodical integration of expert knowledge, which is especially useful when measurable data are limited or several aspects of different importance must be evaluated. The AHP is applied to minimise subjectivity to decide the weighting factors of each variable employed in the analysis. It is commonly employed in multi-criteria decision analysis (MCDA) for spatial planning, particularly when subjective judgements are required to assign relative weights to factors.

### Risk mapping

The risk modelling used two components: susceptibility based on the FR model and vulnerability assessed with AHP. Both quantitative methods included adjustments to the risk index assessment requirements described in Head of National Disaster Management Agency Regulation No. 2 of 2012, including an index scale of 0 to 1 (Van Westen et al. 2011). Results showed that the risk index values ranged from 0.144 to 0.690 and were classified into low, medium and high. The South Jakarta area mainly had medium flood risk, covering 108.64 km^2^ or 74.55% of the total area, while the rest was at low (37.08 km^2^ or 25.45%) and high risk (0.004 km^2^ or 0.003%).

In this study, flood-risk modelling was carried out at a scale of 1:25 000 to align with the South Jakarta Spatial Plan (Figure 5). By comparison, the Provincial Government of Jakarta currently produces flood-risk maps at a coarser scale of 1:100 000. Therefore, the modelling developed in the research can be used to evaluate spatial planning and revise regional spatial plans to incorporate disaster aspects in the future. The current challenge, then, is to incorporate this detailed information into the spatial plan. As the previous study suggests to incorporate risk information into spatial plan, there are four procedures, such as strategic integration, substantive integration, technical integration and procedure integration (Setiawan 2008).

**FIGURE 5a:**
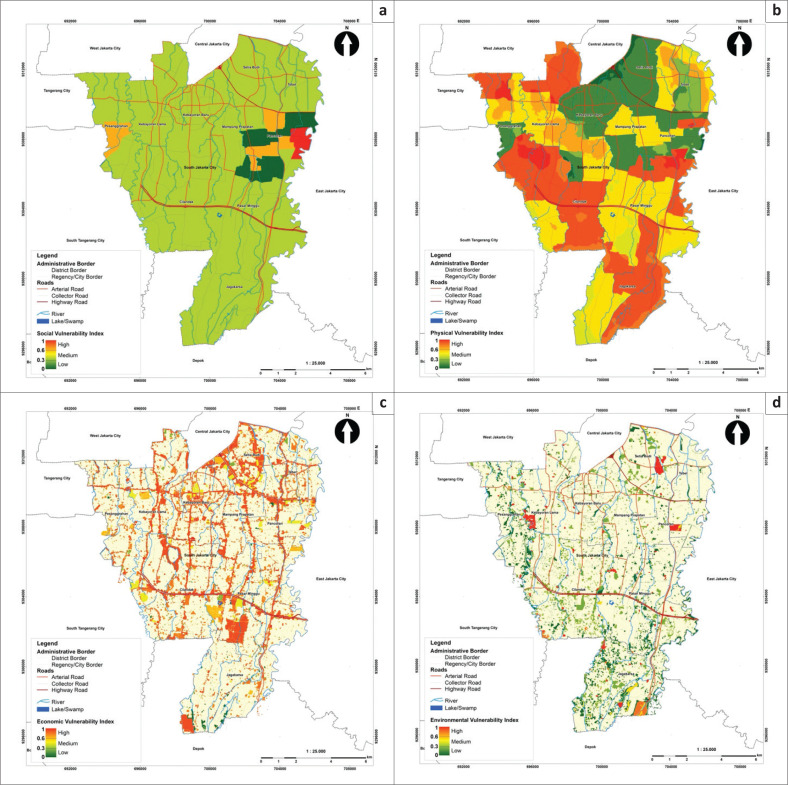
Maps of flood vulnerability levels in South Jakarta; there are six aspects: (a) social vulnerability, (b) physical vulnerability, (c) economic vulnerability, (d) environmental vulnerability.

**FIGURE 5b:**
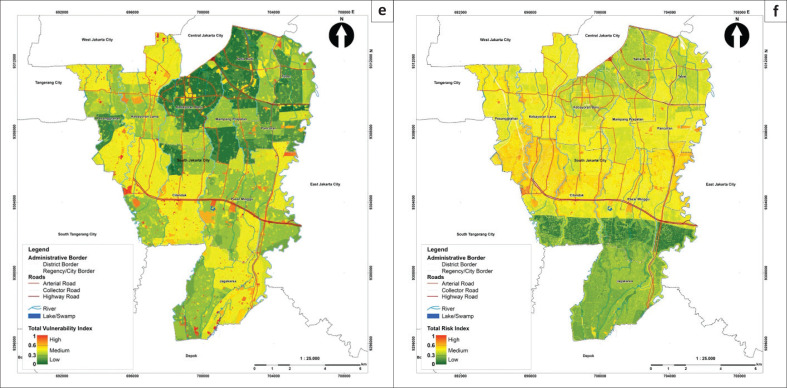
Maps of flood vulnerability levels in South Jakarta; there are six aspects: (e) total vulnerability index and (f) flood-risk index.

According to previous studies, risk information at a 1:25 000 scale allows for substantive integration into a detailed spatial plan. The strategic integration of spatial planning with risk information is mandated by Indonesia’s National Spatial Planning Law No. 26/2007, which requires local governments (urban or city level) to produce risk information at scales of 1:25 000, 1:10 000 and 1:5000. The national law also mentioned that the risk area should be categorised as the spatial pattern of conservation area for risk. Two key components of a detailed spatial plan are the spatial structure and spatial pattern. Incorporating susceptibility, vulnerability and risk information into these components can help prevent further damage and losses in areas exposed to flooding. Within the spatial pattern, such areas can be designated as conservation zones for risk mitigation. However, if nearly 70% of the area is classified as medium risk, an important question arises: how should development proceed under these conditions?

This study shows that high-resolution spatial data can provide useful information to urban planners and decision-makers. Incorporating flood-risk assessments into urban development initiatives will improve disaster risk reduction and strengthen communities in South Jakarta. The spatial plan is a guideline to develop the area. Development permits submitted to the local government are granted only if the application complies with the spatial plan guidelines. Therefore, any proposed development in flood-prone areas must adhere to mitigation regulations. These findings highlight the need for policymakers to prioritise flood risk in urban planning to minimise the socioeconomic impacts of flooding. To effectively reduce flood risk in South Jakarta, practical strategies based on risk mapping must be implemented. Strategies such as improving drainage systems, supporting green infrastructure (e.g. parks, green roofs), flood insurance system (municipality, household, individual, etc.) and encouraging community participation in flood preparedness activities can all help to reduce flood damage. Local governments and stakeholders play an important role in implementing these initiatives, ensuring they are integrated into current urban planning frameworks.

However, it is critical to recognise the limitations of the approach used in this research. While using high-resolution spatial data improves the accuracy of flood-risk assessments, factors such as potential biases in data sources, assumptions made in the FR and AHP models, and spatial data resolution may limit the applicability of these results to other areas of Jakarta or to different contexts within Indonesia. This caution is particularly important in urban environments, where local conditions can significantly influence flood behaviour and risk. Future research should address these limitations by incorporating ground-truth data and examining the heterogeneity of flood hazards across different urban contexts. Additionally, frequent updates to the data – both spatial and temporal – as well as the addition of capacity data could enhance the thoroughness of risk assessments. Building on this study, the FR–AHP framework could be further refined by integrating hydrodynamic modelling and higher-resolution spatial data to improve predictive accuracy and support more adaptive urban planning.

## Conclusion

This research has shown that there are several considerations that need to be taken into account to generate urban flood-risk information. Firstly, each urban area needs to develop detailed risk information; however, many are not fully equipped with adequate statistical and spatial data. Hence, the method selection should be applied according to the existing availability of data and validate the model accordingly. In South Jakarta, flood-risk assessment can be conducted by applying the FR method for hazard or susceptibility modelling, validating the results with ROC–AUC and using the AHP for vulnerability modelling. The flood susceptibility in South Jakarta, Indonesia, is mostly in the medium category (73.171%, 94.101 km^2^), and about one-third of the area has low (22.451%, 28.845 km^2^) and high susceptibility (4.377%, 5.624 km^2^). Among the outputs of flood susceptibility modelling are major flood-conditioning factors, which in this case include rainfall, slope gradient and elevation, land use and distance from the river.

Secondly, the choice of method directly affects the accuracy of the results, which, of course, requires ground verification as well as ongoing monitoring and evaluation. The analytical hierarchical process accommodates the necessity to validate the data through expert judgement and weighting scenarios. The risk information consists of three key components: susceptibility, vulnerability and risk. Each type of analysis can also be conducted using different methods and should be selected based on data availability. Overall, the research revealed that South Jakarta mainly has medium (47%) and low vulnerability (53%). After combining susceptibility with vulnerability, the obtained risk index suggests that the city is generally at medium risk of flooding, covering 108.64 km^2^ of the total area (74.55%), and some parts are at low (37.08 km^2^ or 25.45%) and high risk (0.004 km^2^ or 0.003%). Using spatial data in the form of pixels facilitates and accelerates flood-risk modelling and allows for mapping at a detailed scale. Thirdly, after incorporating the risk information, the spatial plan is a reference document for any development permit; thus, the accuracy and validity of the risk information should be a priority in the method selection.
